# Profile of Pancreatic and Ileal Microbiota in Experimental Acute Pancreatitis

**DOI:** 10.3390/microorganisms11112707

**Published:** 2023-11-04

**Authors:** Mengqi Zhao, Mengyan Cui, Qiaoli Jiang, Jingjing Wang, Yingying Lu

**Affiliations:** 1 Department of Gastroenterology, Shanghai General Hospital, Shanghai Jiao Tong University School of Medicine, Shanghai 201620, China; mqzh0547@sjtu.edu.cn (M.Z.); cuimy0722@sjtu.edu.cn (M.C.); 2 Department of Gastroenterology, Jiading Branch of Shanghai General Hospital, Shanghai Jiao Tong University School of Medicine, Shanghai 201812, China; jiangql_01@163.com; 3 Shanghai Key Laboratory of Pancreatic Diseases, Institute of Translational Medicine, Shanghai General Hospital, Shanghai Jiao Tong University School of Medicine, Shanghai 201620, China

**Keywords:** acute pancreatitis, pancreatic microbiota, ileal microbiota, *Muribaculaceae*, *Dietzia*

## Abstract

Acute pancreatitis (AP) is accompanied by gut microbiota dysbiosis. However, the composition of the pancreatic and ileal microbiota associated with AP is still unknown. This study aims to examine the alterations in the microbial composition of the pancreas and ileum in the context of experimental acute pancreatitis, as well as explore the potential interplay between these two regions. Methods: Caerulein (CAE), caerulein+lipopolysaccharide (CAE+LPS), and L-arginine (ARG) were used to induce AP in mice. The pancreas and ileum were collected for histological study and bacterial 16S rRNA gene sequencing. The results showed microbial structural segregation between the AP and control groups and between ARG and the two CAE groups (CAE, CAE+LPS) in the pancreas and ileum. Taxonomic analysis at the genus level and linear discriminant analysis effect size (LEfSe) at the operational taxonomic units (OTUs) level illustrated that AP mice exhibited a marked increase in the relative abundance of *Muribaculaceae* and a decrease in that of *Dietzia* both in the pancreas and ileum, and a reduction in *Bifidobacterium* only in the ileum; in addition, *Roseburia* was enriched in the two CAE groups in the pancreas and/or ileum, while *Escherichia*–*Shigella* expanded in the pancreas of the ARG group. Spearman correlation analysis between pancreatic and ileal microbiota revealed that the abundance of *Muribaculaceae* and *Dietzia* in the pancreas was related to that in the ileum. These findings demonstrated that caerulein and L-arginine differentially disturbed the pancreatic and ileal microbiota when inducing AP. Furthermore, these findings provide preliminary support for an association between the microbiota of the pancreas and ileum, which could be caused by AP-induced microbial translocation.

## 1. Introduction

Acute pancreatitis (AP) is a common and urgent gastrointestinal disorder that has a rising prevalence worldwide and is a financial burden. It is characterized by premature activation of digestive zymogens within the pancreatic acinar cells and pancreatic self-digestion [1]. About 70–80% of patients develop mild acute pancreatitis (MAP), and about 20–30% develop severe acute pancreatitis (SAP) that has systemic inflammatory response syndrome (SIRS) and multiple organ failure (MOF) [2].

Many clinical and animal studies suggest that gut dysfunction, including gut barrier destruction and gut microbiota dysbiosis, is a crucial event in the AP. In detail, gut barrier dysfunction during AP is associated with an unfavorable prognosis [3,4]; the gut microbiota is often significantly altered in patients with SAP, and dysbiosis of the gut microbiota can induce intestinal mucosal immune dysfunction, increase intestinal permeability, and promote intestinal bacterial translocation [5,6]. Moreover, the small bowel seems to be the major source of enteral bacteria in infected pancreatic necrosis, but not the colon [7].

Many studies applied bacteria culture or qPCR to identify gut bacteria, such as *Escherichia coli*, *Enterobacter aerogenes*, and *Bacillus proteus*, that could translocate to the pancreas during AP [8,9,10]. Bacterial translocation into the pancreas during SAP has been closely associated with pancreatic infection and ultimately leads to serious local complications such as pancreatic necrosis and pancreatic inflammation. Recent studies have shown that intestinal microbiota disorders lead to disruption of iron homeostasis in vivo and that intestinal microbiota metabolites induce iron death in intestinal epithelial cells, promoting intestinal microbiota translocation, which in turn leads to the development of SAP [11,12]. However, few studies have looked into the comprehensive bacterial spectrum, which is translated from the gut to the pancreas during AP.

Thus, the present study is the first to elucidate the ileal and pancreatic microbial changes during AP with bacterial 16S rRNA gene sequencing, compare the different effects on microbiota between caerulein and L-arginine-induced AP, and explore the relationship between pancreatic and ileal microbiota in order to find the bacteria that might migrate from the intestine to the pancreas and are closely related to the occurrence and development of AP.

## 2. Materials and Methods

### 2.1. Mice Experimental Design

Male *C57BL/6* mice weighing 20 to 22 g were purchased from Shanghai SLAC Laboratory Animal Co., Ltd. (Shanghai, China). All mice were housed under specific pathogen-free (SPF) conditions with a room temperature of 23 ± 2 °C, a 12 h shift of the light-dark cycle, and free access to water and mouse chow. All the animal experiments were performed in accordance with the guidelines of the Animal Care and Use Committee of Shanghai Jiao Tong University. All procedures were approved by the Animal Ethics Committee of Shanghai General Hospital.

The mice were randomly divided into four groups, including healthy control (CON), caerulein-induced pancreatitis (CAE), caerulein and LPS-induced pancreatitis (CAE+LPS), and L-arginine-induced pancreatitis (ARG). There were ten mice in the CON group and five mice in each of the other three pancreatitis groups. The AP model was established according to a previous study [13]. For the CAE+LPS group, mice received ten-hourly i.p. injections of 100 μg/kg caerulein and one intraperitoneal injection of LPS (5 mg/kg) immediately after the final injection of caerulein. The mice were anesthetized with chloral hydrate and sacrificed 12 h after the first injection of caerulein. For L-arginine-induced pancreatitis, mice received two-hourly intraperitoneal (i.p.) injections of L-arginine hydrochloride (8%, pH = 7.0, 4.5 g/kg). The mice were anesthetized with chloral hydrate and sacrificed 72 h after the first injection of L-arginine. Furthermore, the mice in the CON group were intraperitoneally injected with normal saline (NS).

### 2.2. Histopathology

Pancreatic and ileal specimens were fixed with 4% paraformaldehyde, dehydrated, embedded with paraffin, cut into 4 μm sections, and stained with hematoxylin and eosin (H&E). The morphologic evaluation was performed by a light microscope (Leica, Wetzlar, Germany) at a magnification of ×200. The histopathological changes to the pancreas, including pancreatic edema, acinar cell necrosis, hemorrhage, and inflammation, were evaluated according to the criteria of Schmidt [14], while the pathological damage to the ileum, including ileal mucosal damage, inflammation, and hemorrhage/congestion, was scored according to Chiu’s standard [15].

### 2.3. Serum Biochemistry

Blood samples from each group were collected and centrifuged at 3000× *g* rpm for 15 min at 4 °C. The serum levels of amylase were determined by using commercial kits in the Advia 2400 chemistry system according to the manufacturer’s protocol (Siemens, Munich, Germany). The serum levels of TNF-α and IL-1β and lipase were measured by enzyme-linked immunosorbent assay (ELISA) (MultiSciences Biotech, Hangzhou, China) according to the manufacturer’s protocols.

### 2.4. Western Blotting

Distal ileal tissues were lysed with radioimmunoprecipitation assay (RIPA) lysis buffer (Epizyme Biotech, Shanghai, China) with a 1% protease inhibitor (Epizyme Biotech, China). The homogenates were settled on ice for 1 h and centrifuged at 10,000× *g* for 10 min at 4 °C, and the supernatants were collected. The extracts were mixed with SDS loading buffer (Yeason, Shanghai, China), and then the solution was heated at 100 °C for 10 min. Ten microliters of the solution were loaded into a 10% SDS-PAGE gel produced by using a PAGE gel fast preparation kit (Epizyme Biotech, China) for electrophoresis, then transferred to polyvinylidene difluoride (PVDF) membranes (Millipore, Burlington, MA, USA). The membranes were blocked with 3% bovine serum albumin (BSA) for 1 h at room temperature and incubated overnight at 4 °C with the antibodies (1:1000): claudin 1 (ABclonal, Shanghai, China); tubulin (Yeason, Shanghai, China). The next day, the membranes were washed with Tris Buffered Saline with Tween-20 (TBST) three times and incubated with peroxidase-conjugated goat anti-rabbit IgG(H1L) (Yeason, China) for 1 h at room temperature. The membranes were washed three times with TBST again. Protein bands were observed using horseradish peroxidase (HRP) substrate peroxide solution (Epizyme Biotech, China) and an Amersham 600 imager (General Electric, Burlington, MA, USA). The density of each band was measured by ImageJ v1.8.0. software.

### 2.5. DNA Extraction PCR Amplification and Sequencing of Bacteria in Pancreas and Ileum

Pancreas and ileum tissues were soaked in 20 μL of 20 mg/mL protease solution and 300 μL of PBS solution at 60 °C overnight. The samples were suspended in 790 μL of sterile lysis buffer (4 M guanidine thiocyanate; 10% N-lauroyl sarcosine; 5% N-lauroyl sarcosine-0.1 M phosphate buffer, pH 8.0) in a 2 mL screw-cap tube containing 1 g glass beads (0.1 mm BioSpec Products, Inc., Valencia, CA, USA). This mixture was vortexed vigorously and then incubated at 70 °C for one hour, followed by bead beating for 10 min at maximum speed. DNA was extracted by following the manufacturer’s instructions for bacterial DNA extraction using the E.Z.N.A.^®^ Stool DNA Kit (Omega Bio-Tek, Inc., Norcross, GA, USA), which included lysis steps and was stored at −20 °C for further analysis.

The V3–V4 region of the bacterial 16S ribosomal RNA gene from each sample was amplified using the bacterial universal primers F1 and R2 (5′-CCTACGGGNGGCWGCAG-3′ and 5′-GACTACHVGGGTATCTAATCC-3′) correspond to positions 341 to 805 in the *Escherichia coli* 16S rRNA gene. PCR reactions were run in an EasyCycler 96 PCR system (Analytik Jena Corp., Upland, CA, USA) using the following program: 3 min of denaturation at 95 °C followed by 21 cycles of 0.5 min at 94 °C (denaturation), 0.5 min for annealing at 58 °C, and 0.5 min at 72 °C (elongation), with a final extension at 72 °C for 5 min.

The products from different samples were indexed and mixed at equal ratios for sequencing by Shanghai Mobio Biomedical Technology Co., Ltd. (Shanghai, China) using the Miseq platform (Illumina Inc., San Diego, CA, USA) according to the manufacturer’s instructions.

### 2.6. Bioinformatics Analysis of Sequencing Data

Clean data was extracted from raw data using USEARCH (version 11.0.667) with the following criteria: (i) Sequences of each sample were extracted using each index with zero mismatches. (ii) Sequences with less than 16 bp overlap were discarded. (iii) The error rate of the overlap greater than 0.1 was discarded. (iv) Sequences less than 400 bp after the merge were discarded. Quality-filtered sequences were clustered into unique sequences and sorted in order of decreasing abundance to identify representative sequences using UPARSE according to the UPARSE OTU analysis pipeline, and singletons were omitted in this step. Operational Taxonomic Units (OTUs) were classified based on 97% similarity after chimeric sequences were removed using UPARSE (version 7.1, http://drive5.com/uparse/ accessed on 8 May 2023) and annotated using the SILVA reference database (SSU138). Alpha diversity metrics (ACE estimator, Chao 1 estimator, Shannon–Wiener diversity index, and Simpson diversity index) were assessed using Mothur v1.42.1. The nonparametric Mann-Whitney U test was used to test for significant differences between the two groups. A comparison of multiple groups was made using a nonparametric Kruskal–Wallis test. Both Bray–Curtis weighted and unweighted UniFrac dissimilarities were calculated in QIIME. Principal coordinate analysis (PCoA) plots and PERMANOVA, which were used to test for statistical significance between the groups using 10,000 permutations, were generated in R (version 3.6.0) package vegan 2.5–7. The linear discriminant analysis (LDA) effect size (LEfSe) was used to detect taxa with differential abundance among groups (lefse 1.1, https://github.com/SegataLab/lefse accessed on 2 June 2023). PICRUSt2 v2.4.1 (https://github.com/picrust/picrust2/wiki accessed on 5 June 2023) was used to predict functional abundances based on 16S rRNA gene sequences.

### 2.7. Statistical Analysis

Data were presented as mean ± standard deviation (SD). Student’s *t*-test, Mann–Whitney test, and Spearman correlation test were performed using SPSS 19.0 software, and a *p*-value of <0.05 was considered statistically significant.

## 3. Results

### 3.1. Caerulein, Caerulein+LPS, and L-Arginine Induced AP in Mice

**Figure 1:**
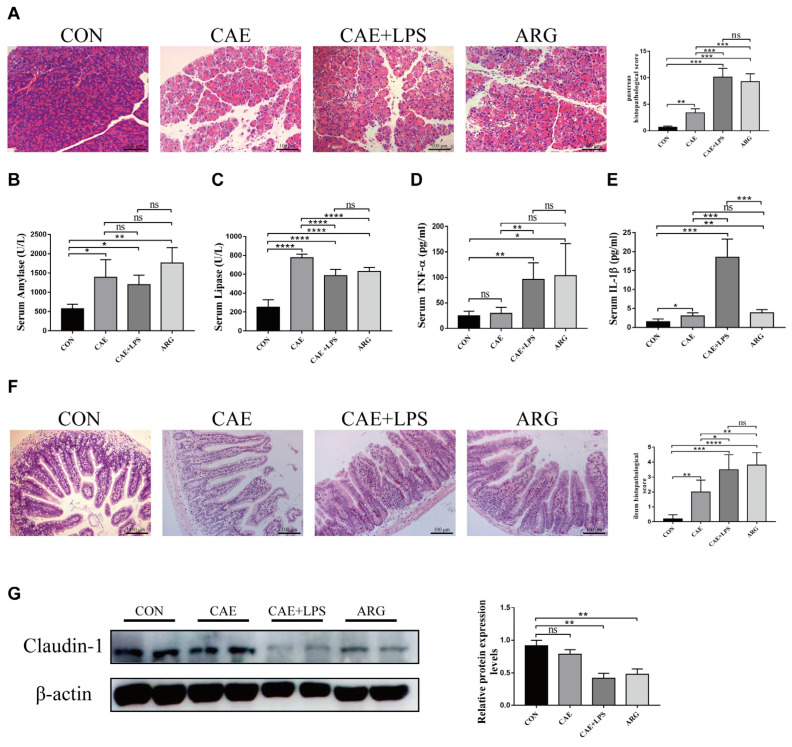
Phenotypes of three AP mouse models induced by Caerulein, Caerulein+LPS, and L-Arginine. (**A**) Histopathological changes in pancreatic samples were observed by HE staining (×200). Scale bar = 100 μm. Pancreatic histopathological scores were evaluated by Schmidt criteria. (**B**,**C**) Serum levels of amylase and lipase. (**D**,**E**) Serum levels of TNF-α and IL-1β. (**F**) Histopathological changes of ileal samples observed by HE staining (×200). Scale bar = 100 μm. (**G**) The protein levels of claudin-1 in the ileum were analyzed by Western blotting. Gels and blots were cropped. Symbol * means *p* < 0.05, ** means *p* < 0.01, *** means *p* < 0.001, **** means *p* < 0.001, ns means *p* > 0.05, Student’s *t*-test.

The three AP models were established by using L-arginine, caerulein, and caerulein+LPS. Pathological pancreatic damage and inflammation were found in all three AP mice, presenting with higher scores of edema, hemorrhage, acinar necrosis, and inflammatory cell infiltration than the control group (Figure 1A). Furthermore, histopathological injury in the pancreas of the CAE+LPS group and ARG group was more aggravated compared with that of the CAE group (Figure 1A). The AP groups exhibited significantly increased serum amylase and lipase levels compared with the CON group (Figure 1B,C). Proinflammatory cytokines IL-1β and TNF-α in the serum were significantly higher in the AP groups than in the control group (Figure 1D,E), suggesting that the AP groups had higher systemic inflammation. Except for the damage and inflammation in the pancreas, AP also induced intestinal injury. Ileal pathological changes, including ileal mucosal damage, inflammation, and hemorrhage/congestion, showed the three AP groups aggravated ileal injury (Figure 1F). In addition, AP induction decreased the expression of claudin-1 in the terminal ileum (Figure 1G), indicating that AP mice had gut barrier dysfunction. The CAE group had less intestinal barrier damage than the other two groups, which was likely owing to the severity difference.

### 3.2. AP Mice Had a More Abundant Microbiota in the Pancreas and Ileum

**Figure 2:**
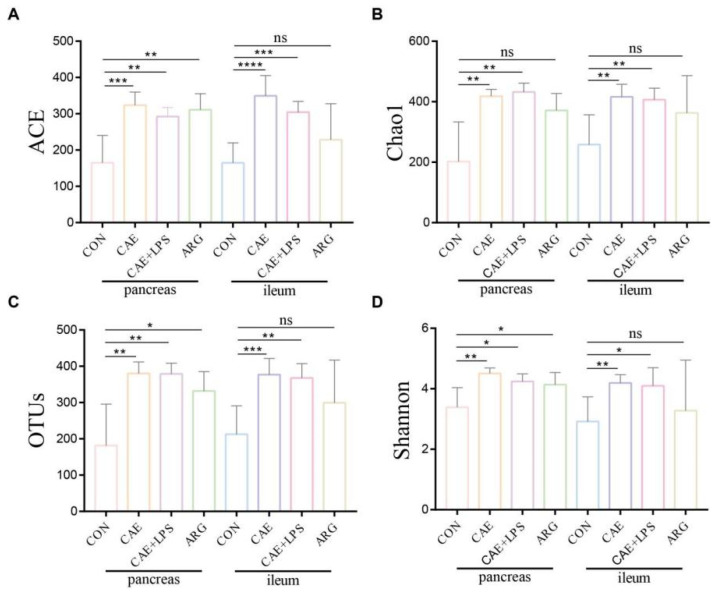
Alpha-diversity in AP and control group both in pancreas and ileum. (**A**–**D**) The estimators of ACE, chao1, OTUs and Shannon of pancreatic and ileal microbiota in each group. Symbol * means *p* < 0.05, ** means *p* < 0.01, *** means *p* < 0.001, **** means *p* < 0.0001, ns means *p* > 0.05, Student’s *t*-test.

A total of 1,117,034 usable sequences were obtained from 50 samples using Illumina high-throughput sequencing technology, with an average of 22,341 sequences per sample. From these, 731 OTUs were generated at a 97% similar level. The rarefaction curves reached a plateau with the current sequencing, and the Shannon diversity estimates of all the samples were stable (Figure S1), indicating that most diversity had already been discovered. The alpha diversities (richness and diversity) of the pancreatic and ileal microbiota were not different in the same group (Figure 2). The three AP groups had significantly higher levels of Shannon indices, Chao1, Ace indices, and OTU numbers than the control group (Figure 2). All the results suggested that AP mice had a more abundant microbiota in both the pancreas and ileum.

### 3.3. AP Changed Pancreatic Microbiota

#### 3.3.1. Differences in Microbiome Compositions in the Pancreas

**Figure 3:**
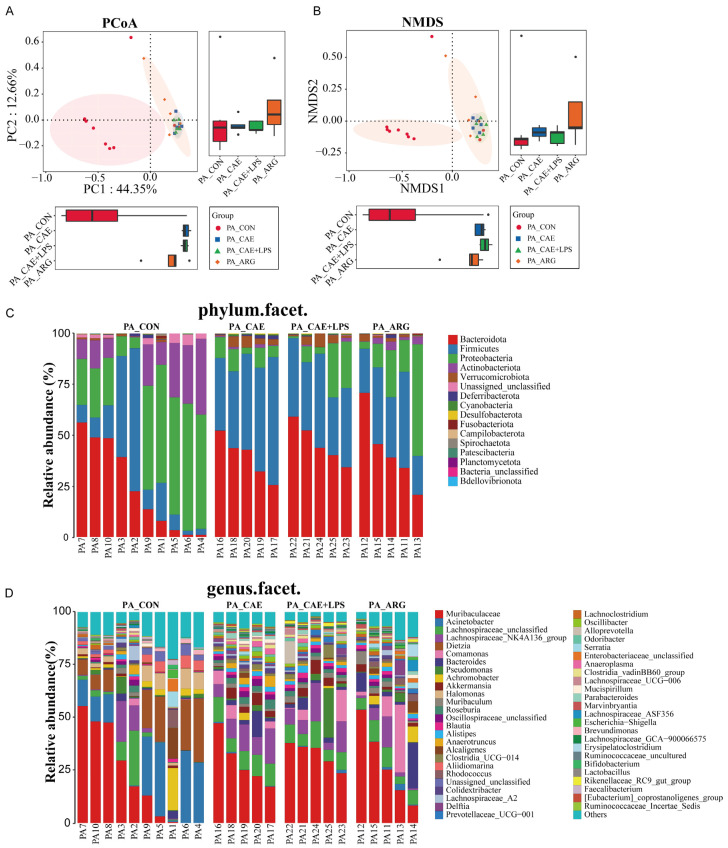
The pancreatic microbiota was changed by AP. (**A**) Principal coordinates analysis plots based on the Bray–Curtis distances showed the differences in the bacterial microbiota between the pancreas of the AP and control groups. The box plots comparing the coordinate positions of the four sets of samples on PC1 and PC2 are shown on the lower left and right, respectively. (**B**) Nonmetric multidimensional scaling analysis based on the Bray–Curtis distances showed the differences among the pancreas of the four groups. Each symbol represents one sample. The box plots comparing the coordinate positions of the four sets of samples on PC1 and PC2 are shown on the lower left and right, respectively. (**C**) Microbiota composition at the phylum level in the pancreas. (**D**) Microbiota composition at the genus level in the pancreas.

Beta diversity analysis was performed to evaluate dissimilarities in pancreatic microbial communities among groups using principal coordinates analysis (PCoA) and non-metric multidimensional scaling (NMDS) analysis based on Bray–Curtis distances (Figure 3A,B). Significant segregation was found between the CON group and AP groups according to PC1, which accounted for 44.35% in PCoA (Figure 3A) and NMDS (Figure 3B) of the total variations of the pancreatic microbiota. To further elucidate the differences in microbial structure, we calculated the relative abundance of bacteria. The microbiota structure at the phylum and genus levels was performed (Figure 3C,D). The pancreatic microbiota was dominated by *Proteobacteria* in the CON group, followed by *Bacteroidota*, *Firmicutes*, and *Actinobacteriota* with proportions of 36.84%, 24.41%, 19.44%, and 15.70%, respectively. Compared with the CON group, increases in *Firmicutes* and *Bacteroidota* abundance and decreases in *Proteobacteria*, *Actinobacteriota*, and Unassigned_unclassified abundance significantly reduced the similarities of the microbiome among the AP groups (Figure 3C). At the genus level, the AP groups had a higher abundance of *Muribaculaceae*, *Lachnosporaceae*, *Akkermansia*, and *Bacteroides* (*p* < 0.05, respectively). The abundance of *Acinetobacter*, *Dietzia*, *Halomonas*, and *Aliidiomarina* in the CON group was significantly higher than in the AP groups (*p* < 0.05, respectively) (Figure 3D).

#### 3.3.2. Differences in the Microbiota of Three AP Groups in the Pancreas

**Figure 4:**
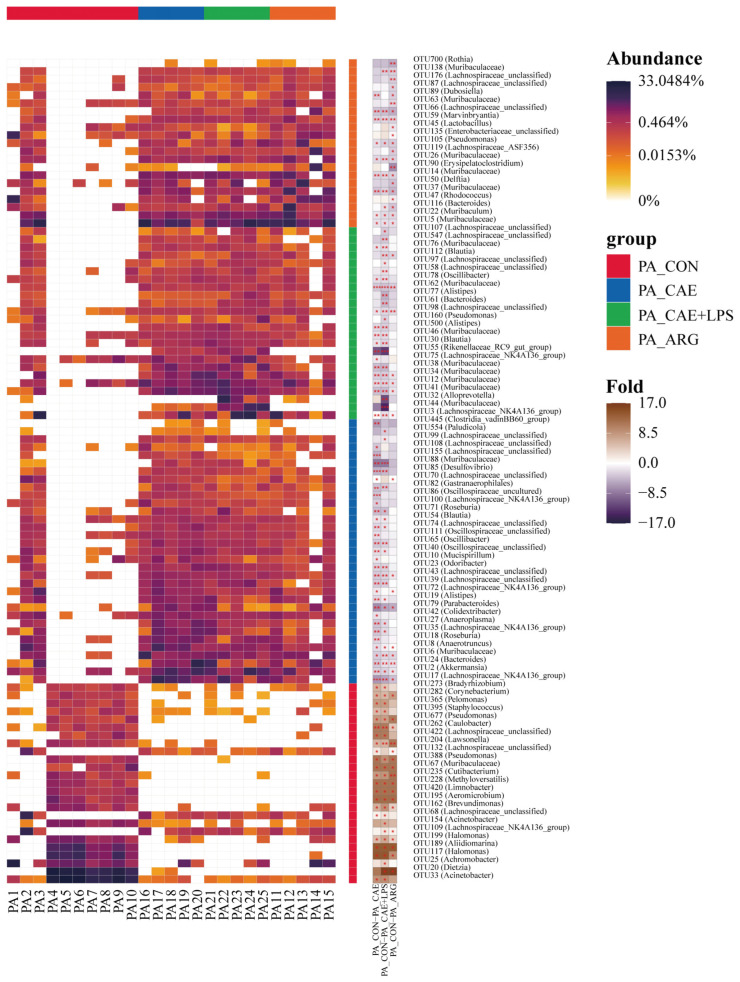
The maximum difference in microbial structures in the pancreas between the CON and AP groups. A heatmap analysis of the microbiomes in the pancreas. Symbol * means *p* < 0.05, ** means *p* < 0.01, *** means *p* < 0.001.

According to LEfSe analysis, the CON and AP groups showed the greatest differences in microbial structure in the pancreas at the OTU level, and then the results of the three groups were pooled and heat map analysis was done (Figure 4). Among these observed OTUs, 59 OTUs were more abundant in the CAE group than the CON group, which belonged to the genera of *Muribaculaceae*, *Lachnospiraceae*_NK4A136_group, *Bacteroides*, *Akkermansia*, and so on (LDA > 3, *p* < 0.05). In comparison, the pancreas of mice in the CAE+LPS group was enriched with 58 OTUs, which belonged to the genera similar to the CAE group. In addition, 39 OTUs significantly with greater abundances of species, including *Muribaculaceae*, *Muribaculum*, *Bacteroides*, *Delftia*, *Lachnospiraceae*_NK4A136_group, were observed in the pancreas of the ARG group than that in the CON group (LDA > 3, *p* < 0.05). And 14 OTUs were richer in the CON group than that in the AP groups, which belonged to the genera of *Corynebacterium*, *Pseudomonas*, *Caulobacter*, *Lawsonella*, and so on (LDA > 3, *p* < 0.05) (Figure S2A–C).

#### 3.3.3. Functional Changes of Three AP Groups in the Pancreas

To characterize the functional alterations in the pancreatic and ileal microbiota of healthy and AP mice, we predicted the functional composition profiles using PICRUSt according to the KEGG database. A comparison between the CON and CAE groups showed that 28 KEGG pathways were enriched in the CON group, including geraniol degradation, the synthesis and degradation of ketone bodies, fatty acid degradation, etc., and 29 pathways were significantly enriched in the CAE group, such as the Biosynthesis of ansamycins, the mRNA surveillance pathway, etc. (Figure S3D) (LDA > 4, *p* < 0.05). The pathways involved in microbial metabolism in the pancreas in the CAE+LPS group were similar to those in the CAE group (Figure S3E) (LDA > 4, *p* < 0.05). A comparison between the CON and ARG groups showed that the metabolism of xenobiotics by cytochrome P450 was significantly enriched in the CON group, and two KEGG pathways, including other glycan degradation and naphthalene degradation, were enriched in the ARG group (Figure S3F).

### 3.4. AP Changed Ileal Microbiota

#### 3.4.1. Differences in Microbiome Compositions in the Ileum

**Figure 5:**
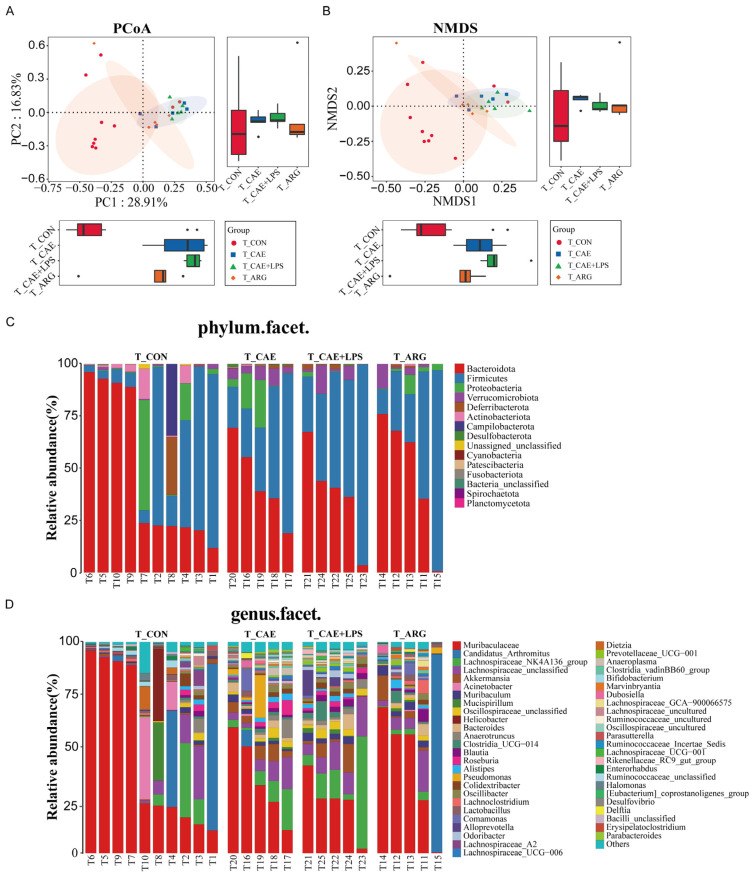
The ileal microbiota was changed by AP. (**A**) Principal coordinates analysis plots based on the Bray–Curtis distances showed the differences in the bacterial microbiota between the ileum of the AP and control groups. The box plots comparing the coordinate positions of the four sets of samples on PC1 and PC2 are shown on the lower left and right, respectively. (**B**) Nonmetric multidimensional scaling analysis based on the Bray–Curtis distances showed the differences among the ileum of the four groups. Each symbol represents one sample. The box plots comparing the coordinate positions of the four sets of samples on PC1 and PC2 are shown on the lower left and right, respectively. (**C**) Microbiota composition at the phylum level in the ileum. (**D**) Microbiota composition at the genus level in the ileum.

The microbiota in the ileum was also modulated by AP. PCoA analysis based on Bray–Curtis distance of ileal microbiota data showed that the first principal component, accounting for 28.91% of the total variance, separated the control group and the three AP groups (Figure 5A). The same results were also shown by NMDS analysis (Figure 5B). The microbiota structure at the phylum and genus levels in the ileum is shown in Figure 5C,D. The ileal microbiota was dominated by *Bacteroidota* in the CON group, followed by *Firmicutes*, *Proteobacteria*, and *Verrucomicrobiota*. At the phylum level, we found that *Deferribacterota*, *Actinobacteriota*, and *Campilobacterota* were significantly lower in the AP groups than in the CON group, while *Verrucomicrobiota* was higher (Figure 5C). At the genus level, the AP groups had a higher abundance of *Muribaculum*, *Oscillospiraceae*_unclassified, and *Bacteroides* (*p* < 0.05, respectively). The abundance of *Helicobacter* and *Dietzia* in the CON group was significantly higher than in the AP groups (*p* < 0.05, respectively) (Figure 5D).

#### 3.4.2. Differences in the Microbiota of Three AP Groups in the Ileum

**Figure 6:**
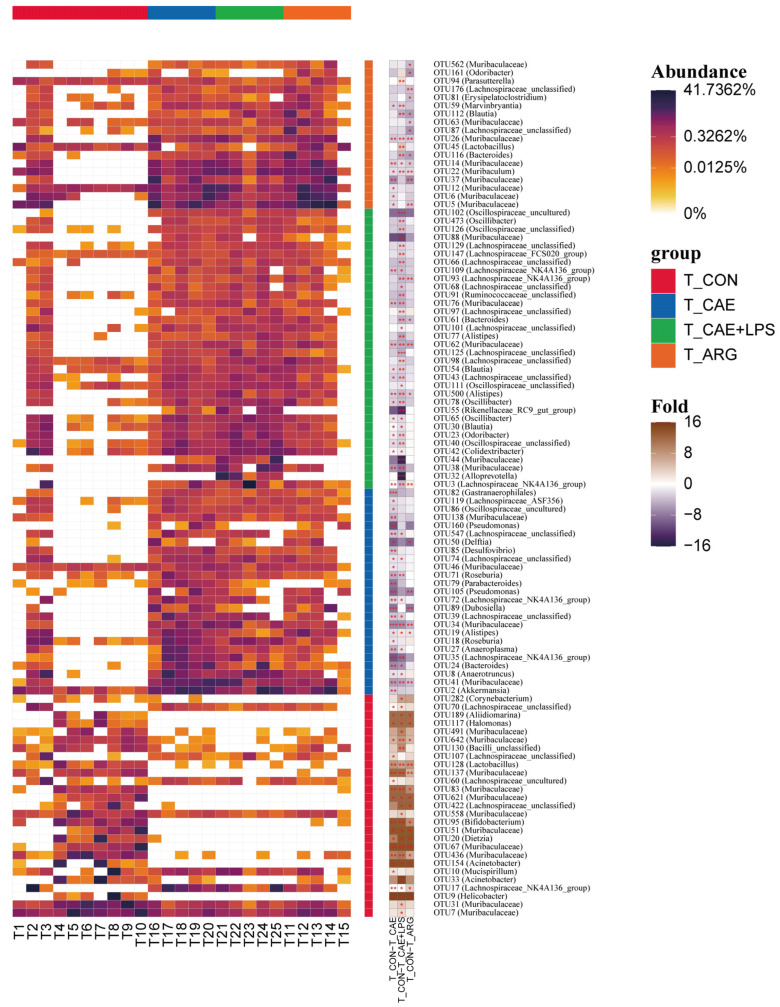
The maximum difference in microbial structures in the ileum between the CON and AP groups. A heatmap analysis of the microbiomes in the ileum. Symbol * means *p* < 0.05, ** means *p* < 0.01, *** means *p* < 0.001.

The largest differences in microbial structure between the CON and AP groups at the OTU level in the ileum were shown according to LEfSe analysis, performing the same thermogram analysis as for the pancreas (Figure 6). Among these observed OTUs, 51 OTUs were more abundant in the CAE group than the CON group, which belonged to the genera of *Akkermansia*, *Muribaculaceae*, *Bacteroides*, and so on (LDA > 3, *p* < 0.05). While the ileum of mice in the CAE+LPS group was enriched with 55 OTUs, which belonged to the genera of *Lachnospiraceae*_NK4A136_group, *Alloprevotella*, *Bacteroides*, etc. (LDA > 3, *p* < 0.05). In addition, 26 OTUs significantly with greater abundances of species, including *Muribaculaceae*, *Muribaculum*, *Bacteroides*, etc., were observed in the ileum of the ARG group than that in the CON group (LDA > 3, *p* < 0.05). And 15 OTUs were richer in the CON group than that in the AP groups, which belonged to the genera of *Aliidiomarina*, *Muribaculaceae*, *Lactobacillus*, *Lachnospiraceae*_NK4A136_group, and so on (LDA > 3, *p* < 0.05) (Figure 6 and Figure S3A–C).

#### 3.4.3. Functional Changes of Three AP Groups in the Ileum

We also compared metabolism pathways in the ileum of the CON and AP groups. naphthalene degradation and butanoate metabolism were significantly higher in the CAE group compared to the control group, while isoflavonoid biosynthesis and nicotinate and nicotinamide metabolism were significantly higher in the control group compared with the CAE group (Figure S3D). In the LPS group, carbon fixation in photosynthetic organisms and cysteine and methionine metabolism were significantly enriched, while lipid metabolism, limonene and pinene degradation, and linoleic acid metabolism were enriched in the control group (Figure S3E). Compared with the control group, the ARG group was enriched in naphthalene degradation and retinol metabolism pathways (Figure S3F).

### 3.5. The Three AP Mice Models Had Distinct Pancreatic and Ileal Microbiota

**Figure 7:**
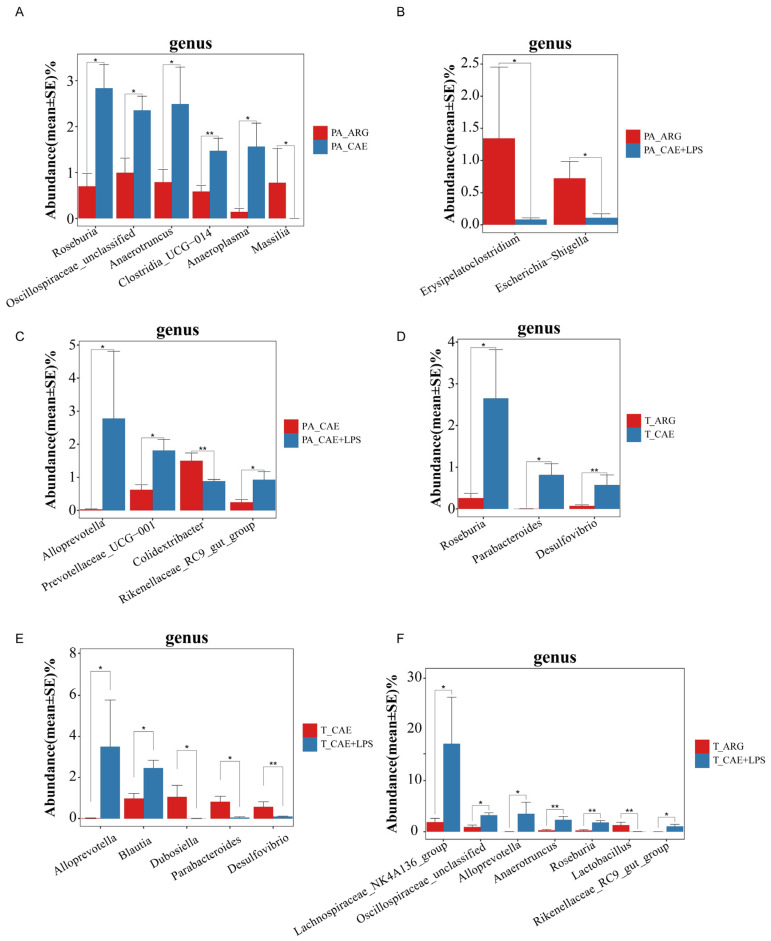
Differences in bacterial composition among the three AP groups in the pancreas and ileum. (**A**) Differences in bacterial composition in the pancreas between the ARG and CAE groups. (**B**) Differences in bacterial composition in the pancreas between the ARG and CAE+LPS groups. (**C**) Differences in bacterial composition in the pancreas between the CAE and CAE+LPS groups. (**D**) Differences in bacterial composition in the ileum between the ARG and CAE groups. (**E**) Differences in bacterial composition in the ileum between the CAE and CAE+LPS groups. (**F**) Differences in bacterial composition in the ileum between the ARG and CAE+LPS groups. Symbol * means *p* < 0.05, ** means *p* < 0.01, Mann–Whitney U tests.

The three AP mice had different pancreatic and ileal microbiota. Microbial diversity analysis in the pancreas showed that the CAE group had the highest microbial diversity, and the microbial diversity in the ARG group was similar to that of the CAE+LPS group (Figure 2 and Figure S1). PCoA and NMDS analyses showed that the overall pancreatic microbial structure of the CAE and CAE+LPS groups was different from that of the ARG group according to PC2, which accounted for 12.66% of the total variance (Figure 3A,B). Compared with the ARG group, the abundance of *Roseburia*, *Oscillospiraceae*_unclassified, *Anaerotruncus*, and so on were enriched, and Massilia was decreased in the CAE group (Figure 7A); the abundance of *Erysipelatoclostridium* and *Escherichia*—*Shigella* was decreased in the pancreas of the LPS group (Figure 7B). The proportions of *Alloprevotella*, *Prevotellaceae*_UCG−001, and *Rikenellaceae*_RC9_gut_group were increased, and the proportion of *Colidextribacter* was decreased in mice of the CAE+LPS group compared to that in the CAE group (Figure 7C).

At the same time, in the ileal microbiota, microbial diversity analysis in the ileal showed that the microbiota α diversity in the CAE group and CAE+LPS group were higher than that in the ARG group, and the level of the ARG group was close to that of the control group (Figure 2 and Figure S1). β-diversity analysis showed that the compositions of bacteria in the CAE group and the CAE+LPS group were similar and deviated slightly from the ARG group (Adonis analysis, *p* = 0.2422, *p* = 0.0072, respectively) according to PC2, which accounted for 16.83% of the total variance (Figure 5A,B). Compared with the ARG group, the CAE group had an increase in *Roseburia*, *Parabacteroides*, and *Desulfovibrio* (Figure 7D), while the LPS group had an increase in *Lachnospiraceae*_NK4A136_group, *Oscillospiraceae*_unclassified, and *Alloprevotella*, and a decrease in *Lactobacillus* (Figure 7F). Compared with the CAE group, the abundance of *Alloprevotella* and *Blautia* in the CAE+LPS group increased significantly, and the abundance of *Dubosiella*, *Parabacteroides*, and *Desulfovibrio* decreased significantly (Figure 7E). All these results suggested that the three AP modeling methods affected the microbiota in both the pancreas and ileum (Figure 3, Figure 4, Figure 5, Figure 6 and Figure 7).

### 3.6. Changed Pancreatic Microbiota Was Associated with the Ileal Microbiota

To further explore the relationship between the pancreatic microbiota and the ileal microbiota of each individual, we performed a correlation analysis between the greatest differences in microbial structure in the pancreas and ileum obtained from the previous LefSe analysis, and the results are shown in Table 1. We found that 14 OTUs were significantly correlated in the pancreas and ileum when restricting rho to >0.8, and these discovered OTUs belong to *Muribaculaceae*, *Halomonas*, *Anaeroplasma*, *Lachnospiraceae*_NK4A136_group, *Rikenellaceae*_RC9_gut_group, *Dietzia*, *Aliidiomarina*, *Delftia*, and *Lachnospiraceae*_unclassified. It was demonstrated that these bacteria were not only maximally different between the CON and AP groups but also significantly correlated in the pancreas and ileum.

**Figure 8:**
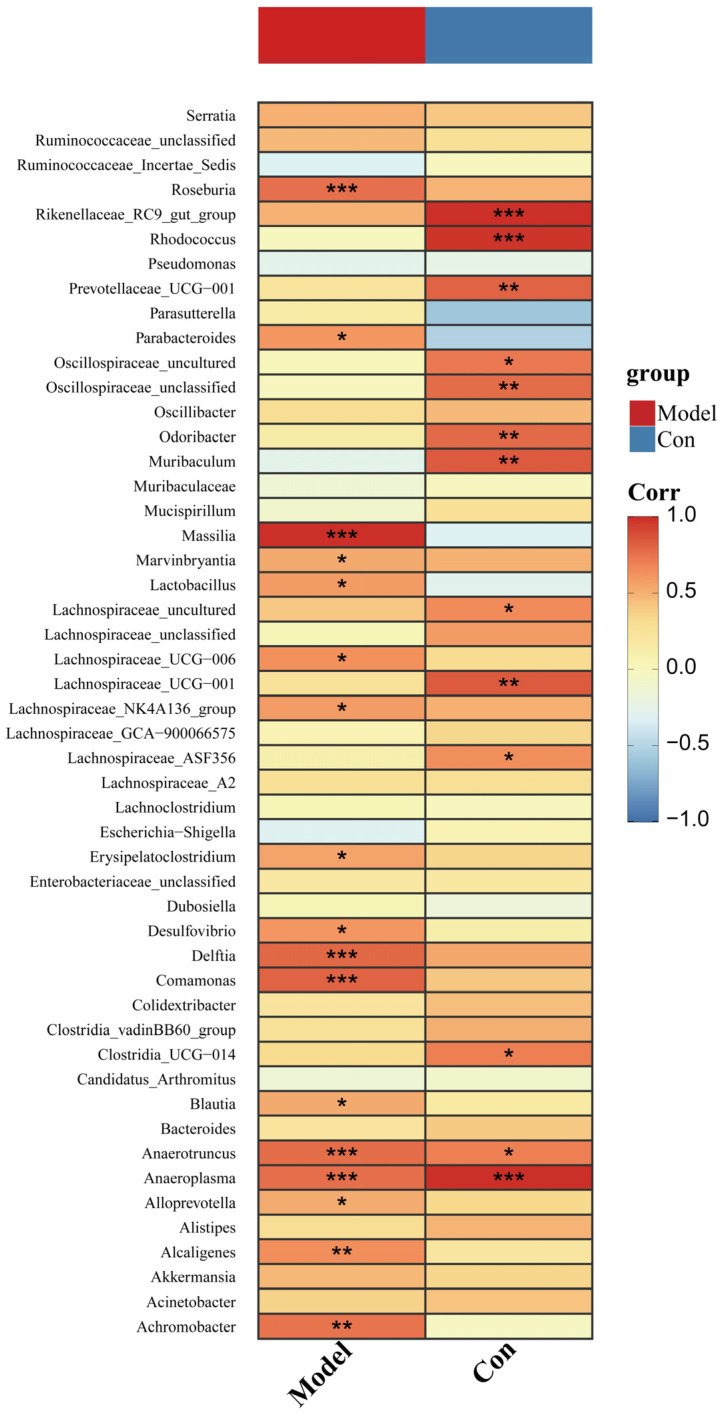
Correlation analysis of changed microbiota in the pancreas and ileum. A Spearman correlation heatmap reflected the correlation between pancreatic microbiota and ileal microbiota. Symbol * means *p* < 0.05, ** means *p* < 0.01, *** means *p* < 0.001, Spearman test.

In addition, we performed the Spearman correlation between the relative abundance of the changed pancreatic microbiota and those of the corresponding bacteria in the ileal with or without induction of AP in mice. We discovered that in the control mice, the microbiota with a significant correlation in pancreas and ileum included *Rikenellaceae*_RC9_gut_group, *Rhodococcus*, *Prevotellaceae*_UCG−001, *Odoribacter*, *Muribaculum*, *Lachnospiraceae*_uncultured, *Lachnospiraceae*_ASF356, *Anaerotruncus*, *Anaeroplasma*, and so on. Most of these bacteria showed an upward trend after induction of AP in mice. However, after AP induction, the genera with a significant correlation between pancreas and ileum included *Roseburia*, *Parabacteroides*, *Marvinbryantia*, *Lachnospiraceae*_NK4A136_group, *Erysipelatoclostridium*, *Desulfovibrio*, *Delftia*, *Comamonas*, *Blautia*, *Anaerotruncus*, *Anaeroplasma*, etc. (Figure 8). The results showed that healthy and AP mice had different relationships between the microbiota from the pancreas and ileum, except for *Anaerotruncus* and *Anaeroplasma*. In addition, these two bacteria were more abundant in the ileum and pancreas during AP.

## 4. Discussion

Although AP exhibits abnormal intestinal permeability and bacterial translocation, the comprehensive bacteria translating from the gut to the pancreas are not clearly understood. Our 16S rRNA gene sequencing-based study in mice confirmed that (1) in the three mice models, AP induced more kinds of bacteria to migrate to the pancreas, including bacteria belonging to *Muribaculaceae*, while reducing the relative abundance of bacteria such as *Dietzia* in the pancreas. These bacteria also changed with the same trend in the ileum. (2) Caerulein and L-arginine differentially disturb the pancreatic and ileal microbiota when inducing AP. In the caerulein-induced AP mice, *Roseburia* was enriched in both the pancreas and ileum, and in the L-arginine-induced AP mice, *escherichia*–*shigella* expanded in the pancreas.

In the present study, caerulein, caerulei+LPS and L-arginine were used to induce AP and ileal injury and inflammation. We found that the bacterial diversity in the pancreas and ileum of mice after induction of AP was significantly higher than that in the control group. Furthermore, previous studies found that *Escherichia*–*Shigella*, *Enterococcus*, and other opportunistic pathogens were overrepresented in AP, whereas the potentially beneficial bacteria *Blautia*, *Lachnospiraceae*, and *Ruminococcaceae* were enriched in the intestinal microbiota of the healthy control [6,16,17,18]. The divergence in our findings on microbiota diversity results can be attributed to the variances in experimental design methodologies employed as compared to previous studies. Distinct differences were observed among various animal species, modeling approaches, and sampling sites. Our study with 16S rRNA gene sequencing identified some different bacteria in the pancreas and ileum. Compared to the control group, *Muribaculaceae*, *Akkermansia*, and *Parabacteroides* were increased, and *Dietzia* was decreased in both the pancreas and the ileum. *Muribaculaceae* is a newly discovered strictly anaerobic gram-negative bacillus, and it is involved in the metabolism of fatty acids like propionic acid [19]. Furthermore, it is controversial about the function of this bacteria; some studies think it is pathogenic and linked to systemic inflammation [20,21], but other studies find it is associated with a reduction in gut inflammation [22,23,24,25]. In this study, *Muribaculaceae* in the pancreas and ileum was enhanced during AP, suggesting that it might be related to the pathology of AP. *Akkermansia* is a commensal bacterium that colonizes the mucosal layer of the intestine and could improve host metabolic dysfunction and reduce inflammation response; thus, it is considered a promising probiotic candidate [26,27]. *Parabacteroides* are reported to produce acetate and ameliorate heparanase-exacerbated acute pancreatitis by reducing neutrophil infiltration [28,29]. *Akkermansia* and *Parabacteroides* were increased in the pancreas and ileum when inducing AP, and this might be due to the protective response of the host against the modeling drug. The gram-positive strain *Dietzia* can grow at low temperatures and is widely distributed in the natural environment. It has been reported to synthesize carotenoids as well as degrade a broad range of n-alkanes [30,31,32]. *Bifidobacterium* were only reduced in the ileum of the three AP groups compared with the healthy control. In the previous study, it had been used with enteral nutrition to treat SAP [33]. It is worth noting that recent research has revealed the significant impact of the oral microbiota on pancreatic disease [34,35].

The mechanisms of AP induced by caerulein and L-arginine are different [36,37], but it is still unclear whether they have different effects on the gut microbiota. This study investigated the effects of different AP induction methods on the microbiota in the gut and pancreas for the first time. According to an unsupervised multivariate analysis, the overall bacterial structure of caerulein groups (CAE, CAE+LPS) and L-arginine groups was separated in the pancreas and ileum. Moreover, *Roseburia* was enriched in the two caerulein groups in the pancreas and/or ileum, while *escherichia*-*shigella* expanded in the pancreas of the L-arginine group. *Roseburia* can prevent intestinal inflammation and regulate barrier homeostasis through the metabolites butyrate and flagellin [38]. An increase in *Roseburia* might protect the host from caerulein-induced AP at the initial stage of the disease. In contrast, *escherichia*–*shigella* can aggravate necrotizing pancreatitis in other studies. KEGG pathway analysis showed that the L-Arg group’s pancreas had increased metabolic pathways, primarily through other glycan degradation and naphthalene degradation. The genus anaplasma was abundant in the pancreas of the L-Arg group, while the *Bacteroidetes phylum* is known for its ability to degrade a variety of complex carbohydrates, a result that corresponds to an increase in the metabolic pathway other glycan degradation [39]. Upon further investigation, we found that the CAE and CAE+LPS groups were involved in comparable pathways in the pancreas, confirming the previous microbiota structure. This result suggests that the microbiota and its functional composition are similar due to the similarity of modeling approaches. Combined with the results of this study, it showed that it might be involved in the L-arginine-induced necrotizing AP [40]. Altogether, despite the disruption of the gut and pancreatic microbiota during the onset of AP, there may still be a compensatory increase in the abundance and translocation of more beneficial bacteria, such as *Roseburia*, in the caerulein-induced AP than in the L-arginine-induced necrotizing AP.

Although previous research has shown that AP disrupts the gut microbiota in humans and animal models, few studies have looked into the correlation between the microbiota in the pancreas and ileum. In this study, Spearman correlation analysis between pancreatic and ileal microbiota revealed the abundances of OTU44 (*Muribaculaceae*), OTU117 (*Halomonas*), OTU27 (*Anaeroplasma*), etc. in the pancreas were related to those in the ileum. These indicated that these bacteria were likely to pass through the intestinal barrier in some way and enter the pancreas from the intestine. Moreover, we discovered that AP could cause different bacterial translocations so that bacteria with a significant correlation in the pancreas and ileum of healthy mice and AP mice were different. A study conducted revealed a significant association between inflammation and the most frequently altered gut bacteria in patients diagnosed with MAP and SAP [5]. This finding suggests that the gut microbiota may play a role in the progression of AP. Consequently, we postulate that the translocation of microbiota could potentially contribute to the exacerbation of acute pancreatitis.

There are still several limitations to this study that need to be addressed. Firstly, the small sample size resulted in significant between-group differences. Secondly, the present study was only an association study and failed to directly prove the existence of microbiota translocation from the ileum to the pancreas.

## 5. Conclusions

In conclusion, we demonstrated that AP induced an imbalance of the pancreatic microbiota, which was closely related to the ileal microbiota, and that cerulein and L-arginine acted on different pancreatic and ileal bacteria. Although the causal relationship and mechanism between these bacteria and AP need to be proved, we have found the microbial markers of AP and can use them as therapeutic targets in the future.

## Figures and Tables

**Table 1:** Correlation analysis between the greatest differences in microbial structure in the pancreas and ileum.

	OTU	Rho	*p*_Value
1	OTU44 (*Muribaculaceae*)	0.934755574	<0.001
2	OTU117 (*Halomonas*)	0.903396145	<0.001
3	OTU27 (*Anaeroplasma*)	0.894354839	<0.001
4	OTU8 (*Anaerotruncus*)	0.890898251	<0.001
5	OTU67 (*Muribaculaceae*)	0.884818826	<0.001
6	OTU3 (*Lachnospiraceae*_NK4A136_group)	0.88152809	<0.001
7	OTU55 (*Rikenellaceae*_RC9_gut_group)	0.875320249	<0.001
8	OTU41 (*Muribaculaceae*)	0.870283019	<0.001
9	OTU35(*Lachnospiraceae*_NK4A136_group)	0.845194922	<0.001
10	OTU20 (*Dietzia*)	0.831554236	<0.001
11	OTU189 (*Aliidiomarina*)	0.825593033	<0.001
12	OTU50 (*Delftia*)	0.809621838	<0.001
13	OTU43 (*Lachnospiraceae*_unclassified)	0.804835266	<0.001
14	OTU88 (*Muribaculaceae*)	0.802774593	<0.001

Note: The Rho representative spearman correlation coefficient, and it is taken to be greater than 0.8.

## Data Availability

The 16S rRNA sequence data generated in this study have been deposited in the Sequence Read Archive database under accession number PRJNA842514. The URL is as follows: https://trace.ncbi.nlm.nih.gov/Traces/sra/ (accessed on 26 May 2023). Now the data has been uploaded but not been public. The data will not be available until paper being accepted.

## Supplementary Materials

The following supporting information can be downloaded at: https://www.mdpi.com/article/10.3390/microorganisms11112707/s1, Figure S1. Rarefaction analysis and Shannon diversity of all samples. (A) The curve of rarefaction analysis. (B) Shannon diversity curve of single sample in each group. Figure S2. Differences in microbiota and functional changes of three AP groups in the pancreas. (A–C) Differences in the microbiota of three AP groups in the pancreas compared to the CON group (LDA > 3, *p* < 0.05). (D–F) Functional changes of three AP groups in the pancreas compared to the CON group (LDA > 4, *p* < 0.05). Figure S3. Differences in microbiota and functional changes of three AP groups in the ileum. (A–C) Differences in the microbiota of three AP groups in the ileum compared to the CON group (LDA > 3, *p* < 0.05). (D–F) Functional changes of three AP groups in the ileum compared to the CON group (LDA > 4, *p* < 0.05).
