# Supplementary material for: Profile of Pancreatic and Ileal Microbiota in Experimental Acute Pancreatitis
Source: Microorganisms. 2023 Nov 4;11(11):2707. doi: 10.3390/microorganisms11112707 (PMC10672832; doi:10.3390/microorganisms11112707)
Supplement: Supplementary file 1 [file microorganisms-11-02707-s001.zip › microorganisms-2647839-supplementary.pdf]

Supplementary Figures

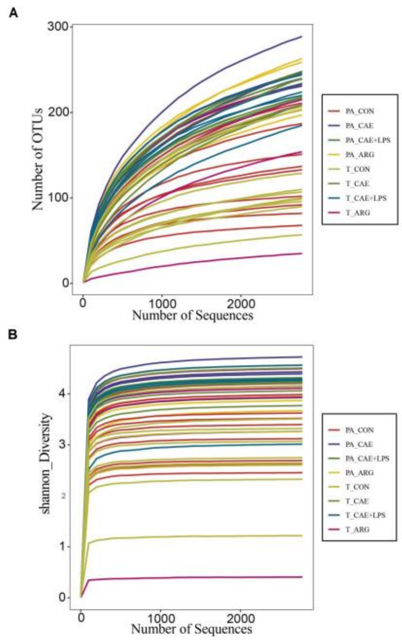

Supplementary Figure S1. Rarefaction analysis and Shannon diversity of all samples. (A) The curve of rarefaction analysis. (B) Shannon diversity curve of single sample in each group.

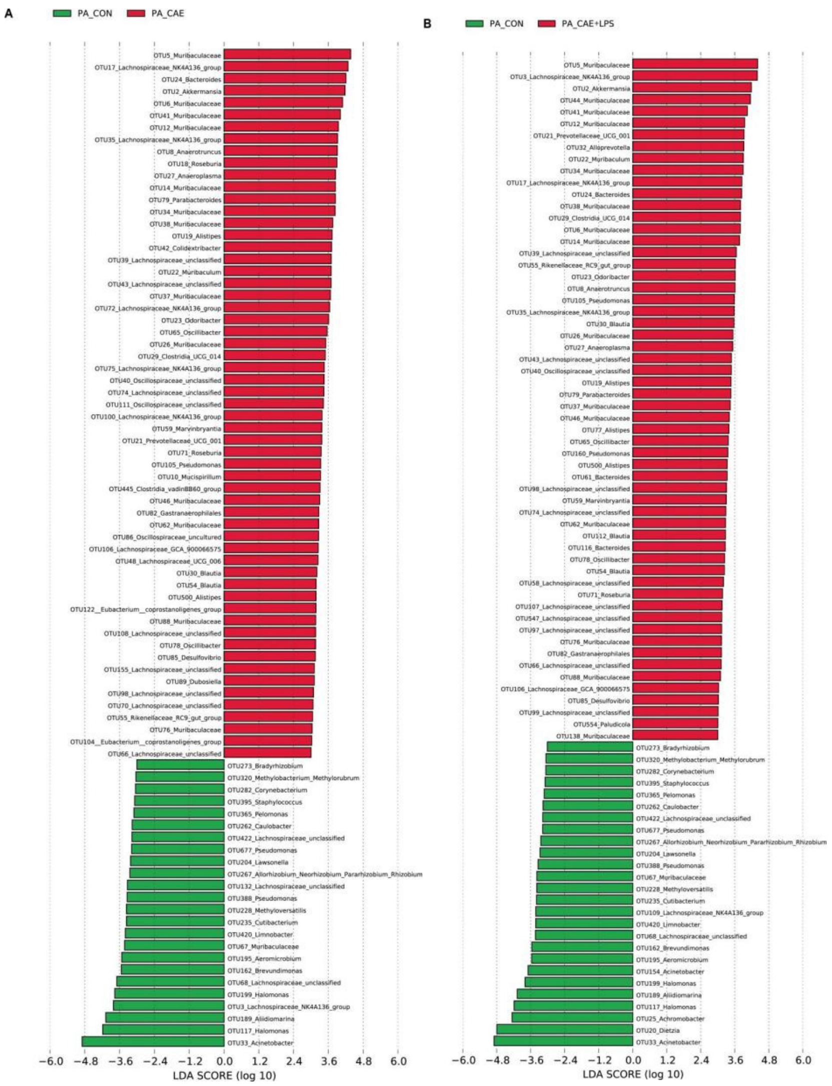
A
PA_CON
PA_CAE
OTU5_Muribaculaceae
OTU17_Lachnospiraceae_NK4A136_group
OTU24_Bacteroides
OTU2_Akkermansia
OTU6_Muribaculaceae
OTU41_Muribaculaceae
OTU12_Muribaculaceae
OTU35_Lachnospiraceae_NK4A136_group
OTU8_Anaerotruncus
OTU18_Roseburia
OTU27_Anaeroplasma
OTU14_Muribaculaceae
OTU79_Parabacteroides
OTU34_Muribaculaceae
OTU38_Muribaculaceae
OTU19_Alistipes
OTU42_Colidextribacter
OTU39_Lachnospiraceae_unclassified
OTU22_Muribaculum
OTU43_Lachnospiraceae_unclassified
OTU37_Muribaculaceae
OTU72_Lachnospiraceae_NK4A136_group
OTU23_Odoribacter
OTU65_Oscillibacter
OTU26_Muribaculaceae
OTU29_Clostridia_UCG_014
OTU75_Lachnospiraceae_NK4A136_group
OTU40_Oscillospiraceae_unclassified
OTU74_Lachnospiraceae_unclassified
OTU111_Oscillospiraceae_unclassified
OTU100_Lachnospiraceae_NK4A136_group
OTU59_Marvinbryantia
OTU21_Prevotellaceae_UCG_001
OTU71_Roseburia
OTU105_Pseudomonas
OTU10_Mucispirillum
OTU445_Clostridia_vadinBB60_group
OTU46_Muribaculaceae
OTU82_Gastranaerophilales
OTU62_Muribaculaceae
OTU86_Oscillospiraceae_uncultured
OTU106_Lachnospiraceae_GCA_900066575
OTU48_Lachnospiraceae_UCG_006
OTU30_Blautia
OTU54_Blautia
OTU500_Alistipes
OTU122__Eubacterium__coprostanoligenes_group
OTU88_Muribaculaceae
OTU108_Lachnospiraceae_unclassified
OTU78_Oscillibacter
OTU85_Desulfovibrio
OTU155_Lachnospiraceae_unclassified
OTU89_Dubosiella
OTU98_Lachnospiraceae_unclassified
OTU70_Lachnospiraceae_unclassified
OTU55_Rikenellaceae_RC9_gut_group
OTU76_Muribaculaceae
OTU104__Eubacterium__coprostanoligenes_group
OTU66_Lachnospiraceae_unclassified
OTU273_Bradyrhizobium
OTU320_Methylobacterium_Methylorubrum
OTU282_Corynebacterium
OTU395_Staphylococcus
OTU365_Pelomonas
OTU262_Caulobacter
OTU422_Lachnospiraceae_unclassified
OTU677_Pseudomonas
OTU204_Lawsonella
OTU267_Allorhizobium_Neorhizobium_Pararhizobium_Rhizobium
OTU132_Lachnospiraceae_unclassified
OTU388_Pseudomonas
OTU228_Methyloversatilis
OTU235_Cutibacterium
OTU420_Limnobacter
OTU67_Muribaculaceae
OTU195_Aeromicrobium
OTU162_Brevundimonas
OTU68_Lachnospiraceae_unclassified
OTU199_Halomonas
OTU3_Lachnospiraceae_NK4A136_group
OTU189_Aliidiomarina
OTU117_Halomonas
OTU33_Acinetobacter
−6.0 −4.8 −3.6 −2.4 −1.2 0.0 1.2 2.4 3.6 4.8 6.0
LDA SCORE (log 10)
B
PA_CON
PA_CAE+LPS
OTU5_Muribaculaceae
OTU3_Lachnospiraceae_NK4A136_group
OTU2_Akkermansia
OTU44_Muribaculaceae
OTU41_Muribaculaceae
OTU12_Muribaculaceae
OTU21_Prevotellaceae_UCG_001
OTU32_Alloprevotella
OTU22_Muribaculum
OTU34_Muribaculaceae
OTU17_Lachnospiraceae_NK4A136_group
OTU24_Bacteroides
OTU38_Muribaculaceae
OTU29_Clostridia_UCG_014
OTU6_Muribaculaceae
OTU14_Muribaculaceae
OTU39_Lachnospiraceae_unclassified
OTU55_Rikenellaceae_RC9_gut_group
OTU23_Odoribacter
OTU8_Anaerotruncus
OTU105_Pseudomonas
OTU35_Lachnospiraceae_NK4A136_group
OTU30_Blautia
OTU26_Muribaculaceae
OTU27_Anaeroplasma
OTU43_Lachnospiraceae_unclassified
OTU40_Oscillospiraceae_unclassified
OTU19_Alistipes
OTU79_Parabacteroides
OTU37_Muribaculaceae
OTU46_Muribaculaceae
OTU77_Alistipes
OTU65_Oscillibacter
OTU160_Pseudomonas
OTU500_Alistipes
OTU61_Bacteroides
OTU98_Lachnospiraceae_unclassified
OTU59_Marvinbryantia
OTU74_Lachnospiraceae_unclassified
OTU62_Muribaculaceae
OTU112_Blautia
OTU116_Bacteroides
OTU78_Oscillibacter
OTU54_Blautia
OTU58_Lachnospiraceae_unclassified
OTU71_Roseburia
OTU107_Lachnospiraceae_unclassified
OTU547_Lachnospiraceae_unclassified
OTU97_Lachnospiraceae_unclassified
OTU76_Muribaculaceae
OTU82_Gastranaerophilales
OTU66_Lachnospiraceae_unclassified
OTU88_Muribaculaceae
OTU106_Lachnospiraceae_GCA_900066575
OTU85_Desulfovibrio
OTU99_Lachnospiraceae_unclassified
OTU554_Paludicola
OTU138_Muribaculaceae
OTU273_Bradyrhizobium
OTU320_Methylobacterium_Methylorubrum
OTU282_Corynebacterium
OTU395_Staphylococcus
OTU365_Pelomonas
OTU262_Caulobacter
OTU422_Lachnospiraceae_unclassified
OTU677_Pseudomonas
OTU267_Allorhizobium_Neorhizobium_Pararhizobium_Rhizobium
OTU204_Lawsonella
OTU388_Pseudomonas
OTU67_Muribaculaceae
OTU228_Methyloversatilis
OTU235_Cutibacterium
OTU109_Lachnospiraceae_NK4A136_group
OTU420_Limnobacter
OTU68_Lachnospiraceae_unclassified
OTU162_Brevundimonas
OTU195_Aeromicrobium
OTU154_Acinetobacter
OTU199_Halomonas
OTU189_Aliidiomarina
OTU117_Halomonas
OTU25_Achromobacter
OTU20_Dietzia
OTU33_Acinetobacter
−6.0 −4.8 −3.6 −2.4 −1.2 0.0 1.2 2.4 3.6 4.8 6.0
LDA SCORE (log 10)

C

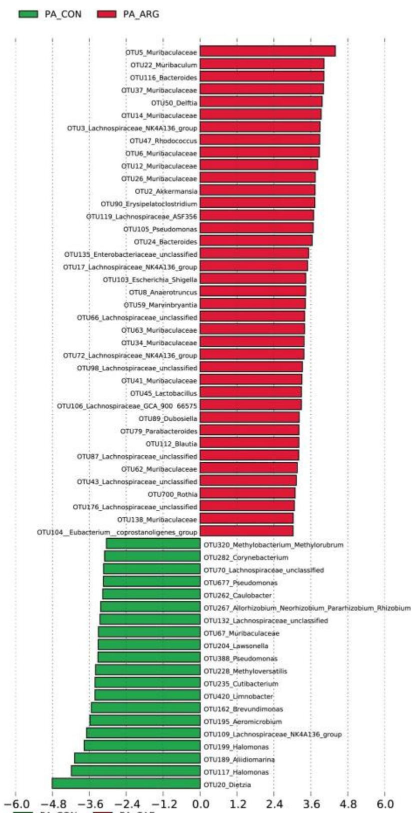
PA_CON
PA_ARG
OTU5_Muribaculaceae
OTU22_Muribaculum
OTU116_Bacteroides
OTU37_Muribaculaceae
OTU50_Delftia
OTU14_Muribaculaceae
OTU3_Lachnospiraceae_NK4A136_group
OTU47_Rhodococcus
OTU6_Muribaculaceae
OTU12_Muribaculaceae
OTU26_Muribaculaceae
OTU2_Akkermansia
OTU90_Erysipelatoclostridium
OTU119_Lachnospiraceae_ASF356
OTU105_Pseudomonas
OTU24_Bacteroides
OTU135_Enterobacteriaceae_unclassified
OTU17_Lachnospiraceae_NK4A136_group
OTU103_Escherichia_Shigella
OTU8_Anaerotruncus
OTU59_Marvinbryantia
OTU66_Lachnospiraceae_unclassified
OTU63_Muribaculaceae
OTU34_Muribaculaceae
OTU72_Lachnospiraceae_NK4A136_group
OTU98_Lachnospiraceae_unclassified
OTU41_Muribaculaceae
OTU45_Lactobacillus
OTU106_Lachnospiraceae_GCA_900 66575
OTU89_Dubosiella
OTU79_Parabacteroides
OTU112_Blautia
OTU87_Lachnospiraceae_unclassified
OTU62_Muribaculaceae
OTU43_Lachnospiraceae_unclassified
OTU700_Rothia
OTU176_Lachnospiraceae_unclassified
OTU138_Muribaculaceae
OTU104_Eubacterium_coprostanoligenes_group
OTU320_Methylobacterium_Methylorubrum
OTU282_Corynebacterium
OTU70_Lachnospiraceae_unclassified
OTU677_Pseudomonas
OTU262_Caulobacter
OTU267_Allorhizobium_Neorhizobium_Pararhizobium_Rhizobium
OTU132_Lachnospiraceae_unclassified
OTU67_Muribaculaceae
OTU204_Lawsonella
OTU388_Pseudomonas
OTU228_Methyloversatilis
OTU235_Cutibacterium
OTU420_Limnobacter
OTU162_Brevundimonas
OTU195_Aeromicrobium
OTU109_Lachnospiraceae_NK4A136_group
OTU199_Halomonas
OTU189_Aliidiomarina
OTU117_Halomonas
OTU20_Dietzia
−6.0 −4.8 −3.6 −2.4 −1.2 0.0 1.2 2.4 3.6 4.8 6.0

D

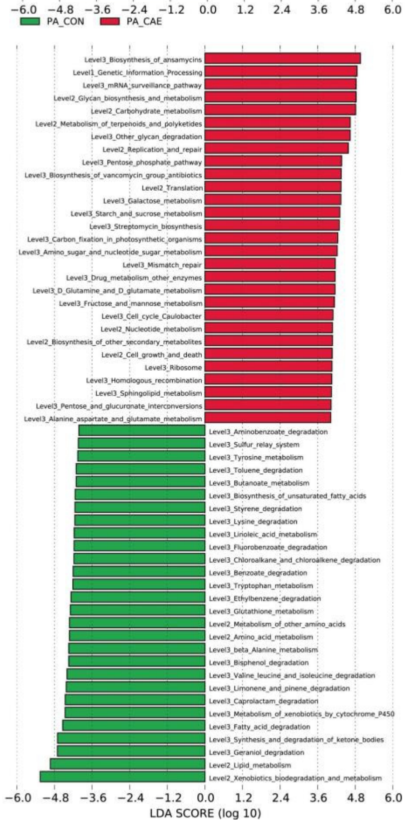
PA_CON
PA_CAE
Level3_Biosynthesis_of_ansamycins
Level1_Genetic_Information_Processing
Level3_mRNA_surveillance_pathway
Level2_Glycan_biosynthesis_and_metabolism
Level2_Carbohydrate_metabolism
Level2_Metabolism_of_terpenoids_and_polyketides
Level3_Other_glycan_degradation
Level2_Replication_and_repair
Level3_Pentose_phosphate_pathway
Level3_Biosynthesis_of_vancomycin_group_antibiotics
Level2_Translation
Level3_Galactose_metabolism
Level3_Starch_and_sucrose_metabolism
Level3_Streptomycin_biosynthesis
Level3_Carbon_fixation_in_photosynthetic_organisms
Level3_Amino_sugar_and_nucleotide_sugar_metabolism
Level3_Mismatch_repair
Level3_Drug_metabolism_other_enzymes
Level3_D_Glutamine_and_D_glutamate_metabolism
Level3_Fructose_and_mannose_metabolism
Level3_Cell_cycle_Caulobacter
Level2_Nucleotide_metabolism
Level2_Biosynthesis_of_other_secondary_metabolites
Level2_Cell_growth_and_death
Level3_Ribosome
Level3_Homologous_recombination
Level3_Sphingolipid_metabolism
Level3_Pentose_and_glucuronate_interconversions
Level3_Alanine_aspartate_and_glutamate_metabolism
Level3_Aminobenzoate_degradation
Level3_Sulfur_relay_system
Level3_Tyrosine_metabolism
Level3_Toluene_degradation
Level3_Butanoate_metabolism
Level3_Biosynthesis_of_unsaturated_fatty_acids
Level3_Styrene_degradation
Level3_Lysine_degradation
Level3_Linoleic_acid_metabolism
Level3_Fluorobenzoate_degradation
Level3_Chloroalkane_and_chloroalkene_degradation
Level3_Benzoate_degradation
Level3_Tryptophan_metabolism
Level3_Ethylbenzene_degradation
Level3_Glutathione_metabolism
Level2_Metabolism_of_other_amino_acids
Level2_Amino_acid_metabolism
Level3_beta_Alanine_metabolism
Level3_Bisphenol_degradation
Level3_Valine_leucine_and_isoleucine_degradation
Level3_Limonene_and_pinene_degradation
Level3_Caprolactam_degradation
Level3_Metabolism_of_xenobiotics_by_cytochrome_P450
Level3_Fatty_acid_degradation
Level3_Synthesis_and_degradation_of_ketone_bodies
Level3_Geraniol_degradation
Level2_Lipid_metabolism
Level2_Xenobiotics_biodegradation_and_metabolism
−6.0 −4.8 −3.6 −2.4 −1.2 0.0 1.2 2.4 3.6 4.8 6.0
LDA SCORE (log 10)

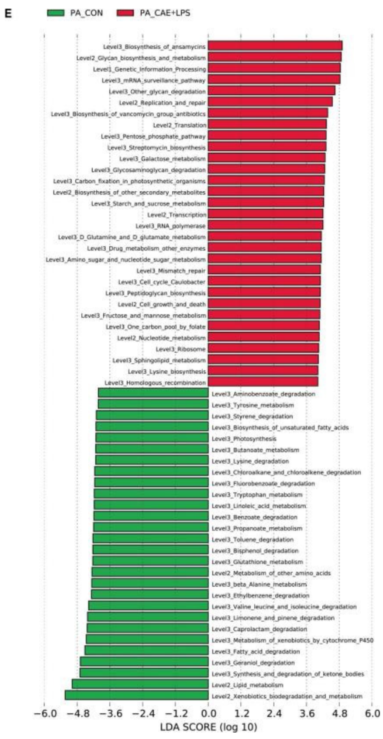

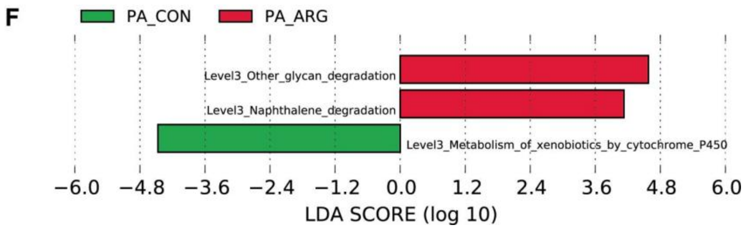

Supplementary Figure S2. Differences in microbiota and functional changes of three AP groups in the pancreas. (A-C) Differences in the microbiota of three AP groups in the pancreas compared to the CON group (LDA＞3, P＜0.05). (D-F) Functional changes of three AP groups in the pancreas compared to the CON group (LDA＞4, P＜0.05).

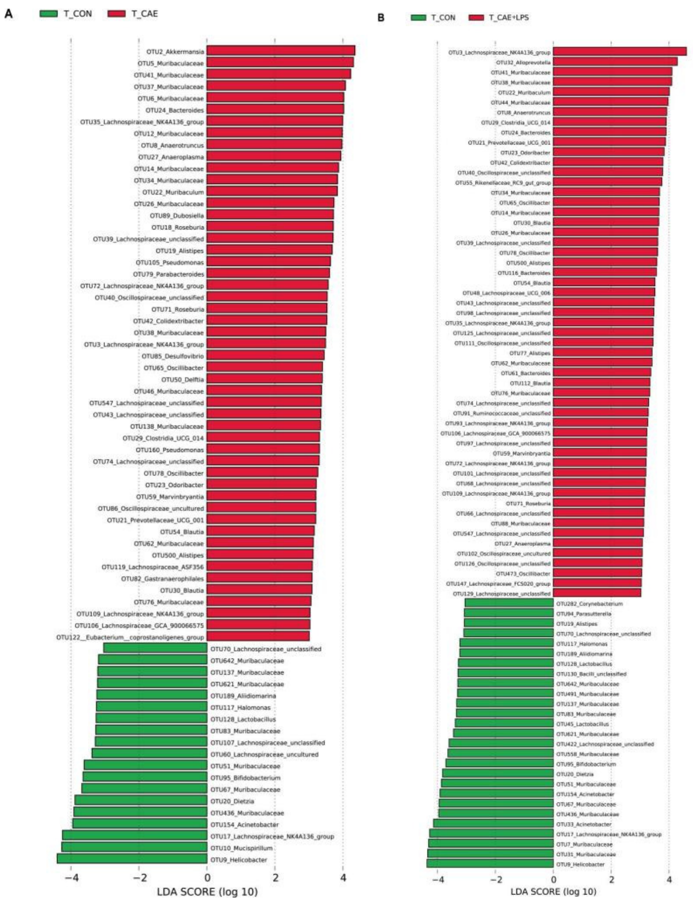
A
T_CON
T_CAE
OTU2_Akkermansia
OTU5_Muribaculaceae
OTU41_Muribaculaceae
OTU37_Muribaculaceae
OTU6_Muribaculaceae
OTU24_Bacteroides
OTU35_Lachnospiraceae_NK4A136_group
OTU12_Muribaculaceae
OTU8_Anaerotruncus
OTU27_Anaeroplasma
OTU14_Muribaculaceae
OTU34_Muribaculaceae
OTU22_Muribaculum
OTU26_Muribaculaceae
OTU89_Dubosiella
OTU18_Roseburia
OTU39_Lachnospiraceae_unclassified
OTU19_Alistipes
OTU105_Pseudomonas
OTU79_Parabacteroides
OTU72_Lachnospiraceae_NK4A136_group
OTU40_Oscillospiraceae_unclassified
OTU71_Roseburia
OTU42_Colidextribacter
OTU38_Muribaculaceae
OTU3_Lachnospiraceae_NK4A136_group
OTU85_Desulfovibrio
OTU65_Oscillibacter
OTU50_Delftia
OTU46_Muribaculaceae
OTU547_Lachnospiraceae_unclassified
OTU43_Lachnospiraceae_unclassified
OTU138_Muribaculaceae
OTU29_Clostridia_UCG_014
OTU160_Pseudomonas
OTU74_Lachnospiraceae_unclassified
OTU78_Oscillibacter
OTU23_Odoribacter
OTU59_Marvinbryantia
OTU86_Oscillospiraceae_uncultured
OTU21_Prevotellaceae_UCG_001
OTU54_Blautia
OTU62_Muribaculaceae
OTU500_Alistipes
OTU119_Lachnospiraceae_ASF356
OTU82_Gastranaerophilales
OTU30_Blautia
OTU76_Muribaculaceae
OTU109_Lachnospiraceae_NK4A136_group
OTU106_Lachnospiraceae_GCA_900066575
OTU122__Eubacterium__coprostanoligenes_group
OTU70_Lachnospiraceae_unclassified
OTU642_Muribaculaceae
OTU137_Muribaculaceae
OTU621_Muribaculaceae
OTU189_Aliidiomarina
OTU117_Halomonas
OTU128_Lactobacillus
OTU83_Muribaculaceae
OTU107_Lachnospiraceae_unclassified
OTU60_Lachnospiraceae_uncultured
OTU51_Muribaculaceae
OTU95_Bifidobacterium
OTU67_Muribaculaceae
OTU20_Dietzia
OTU436_Muribaculaceae
OTU154_Acinetobacter
OTU17_Lachnospiraceae_NK4A136_group
OTU10_Mucispirillum
OTU9_Helicobacter
−4
−2
0
2
4
LDA SCORE (log 10)
B
T_CON
T_CAE+LPS
OTU3_Lachnospiraceae_NK4A136_group
OTU32_Alloprevotella
OTU41_Muribaculaceae
OTU38_Muribaculaceae
OTU22_Muribaculum
OTU44_Muribaculaceae
OTU8_Anaerotruncus
OTU29_Clostridia_UCG_014
OTU24_Bacteroides
OTU21_Prevotellaceae_UCG_001
OTU23_Odoribacter
OTU42_Colidextribacter
OTU40_Oscillospiraceae_unclassified
OTU55_Rikenellaceae_RC9_gut_group
OTU34_Muribaculaceae
OTU65_Oscillibacter
OTU14_Muribaculaceae
OTU30_Blautia
OTU26_Muribaculaceae
OTU39_Lachnospiraceae_unclassified
OTU78_Oscillibacter
OTU500_Alistipes
OTU116_Bacteroides
OTU54_Blautia
OTU48_Lachnospiraceae_UCG_006
OTU43_Lachnospiraceae_unclassified
OTU98_Lachnospiraceae_unclassified
OTU35_Lachnospiraceae_NK4A136_group
OTU125_Lachnospiraceae_unclassified
OTU111_Oscillospiraceae_unclassified
OTU77_Alistipes
OTU62_Muribaculaceae
OTU61_Bacteroides
OTU112_Blautia
OTU76_Muribaculaceae
OTU74_Lachnospiraceae_unclassified
OTU91_Ruminococcaceae_unclassified
OTU93_Lachnospiraceae_NK4A136_group
OTU106_Lachnospiraceae_GCA_900066575
OTU97_Lachnospiraceae_unclassified
OTU59_Marvinbryantia
OTU72_Lachnospiraceae_NK4A136_group
OTU101_Lachnospiraceae_unclassified
OTU68_Lachnospiraceae_unclassified
OTU109_Lachnospiraceae_NK4A136_group
OTU71_Roseburia
OTU66_Lachnospiraceae_unclassified
OTU88_Muribaculaceae
OTU547_Lachnospiraceae_unclassified
OTU27_Anaeroplasma
OTU102_Oscillospiraceae_uncultured
OTU126_Oscillospiraceae_unclassified
OTU473_Oscillibacter
OTU147_Lachnospiraceae_FCS020_group
OTU129_Lachnospiraceae_unclassified
OTU282_Corynebacterium
OTU94_Parasutterella
OTU19_Alistipes
OTU70_Lachnospiraceae_unclassified
OTU117_Halomonas
OTU189_Aliidiomarina
OTU128_Lactobacillus
OTU130_Bacilli_unclassified
OTU642_Muribaculaceae
OTU491_Muribaculaceae
OTU137_Muribaculaceae
OTU83_Muribaculaceae
OTU45_Lactobacillus
OTU621_Muribaculaceae
OTU422_Lachnospiraceae_unclassified
OTU558_Muribaculaceae
OTU95_Bifidobacterium
OTU20_Dietzia
OTU51_Muribaculaceae
OTU154_Acinetobacter
OTU67_Muribaculaceae
OTU436_Muribaculaceae
OTU33_Acinetobacter
OTU17_Lachnospiraceae_NK4A136_group
OTU7_Muribaculaceae
OTU31_Muribaculaceae
OTU9_Helicobacter
−4
−2
0
2
4
LDA SCORE (log 10)

C

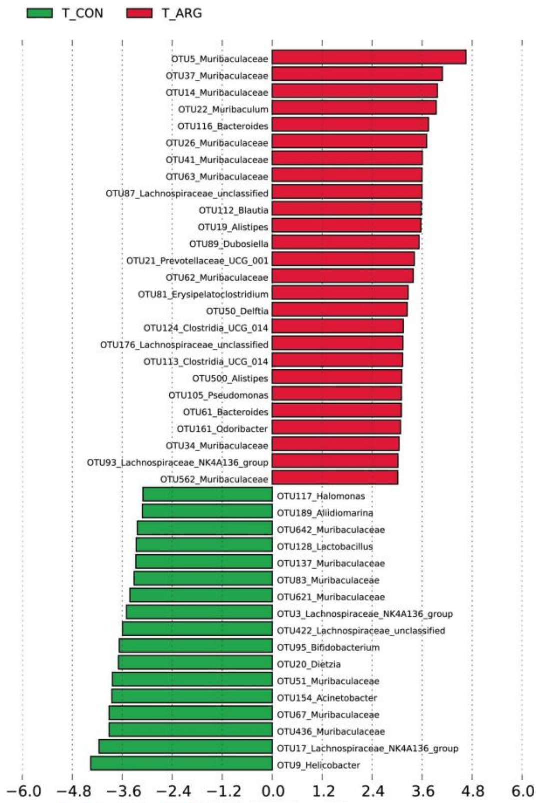

D

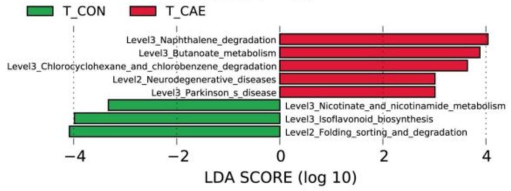

E

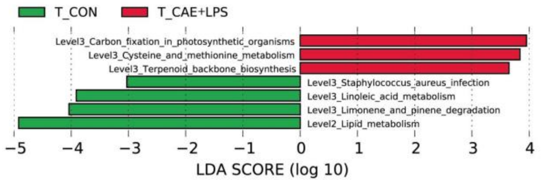

F

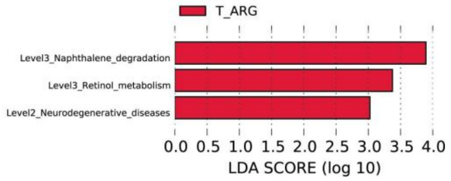

Supplementary Figure S3. Differences in microbiota and functional changes of three AP groups in the ileum. (A-C) Differences in the microbiota of three AP groups in the ileum compared to the CON group (LDA>3, P<0.05). (D-F) Functional changes of three AP groups in the ileum compared to the CON group (LDA>4, P<0.05).
